# Aortic Thrombosis and Ischemic Stroke With Hemorrhagic Conversion in a Patient With Remote COVID-19 Infection: A Treatment Dilemma

**DOI:** 10.7759/cureus.25272

**Published:** 2022-05-24

**Authors:** Maria Riasat, Arshan Khan, Moiz Ehtesham, Syed Farrukh Mustafa, Natasha Qureshi

**Affiliations:** 1 Internal Medicine, Icahn School of Medicine at Mount Sinai Beth Israel, New York, USA; 2 Internal Medicine, Ascension St. John Hospital, Detroit, USA; 3 Internal Medicine, Albany Medical College, Albany, USA; 4 Cardiology, Icahn School of Medicine at Mount Sinai Beth Israel, New York, USA

**Keywords:** in hospital cardiac arrest, post covid-19 sequelae, ischemic cerebrovascular disease, aortic mobile thrombus, hypercoaguable

## Abstract

Coronavirus disease 2019 (COVID-19) is primarily known to affect the lungs; however, several studies indicate that it can be a multisystem disease. There is documentation detailing different sequelae of severe acute respiratory syndrome coronavirus 2 (SARS-CoV-2). Patients affected with this virus have been seen to develop a hypercoagulable state leading to systemic thrombosis in some cases or embolism leading to catastrophic outcomes in others. Data regarding anticoagulation in these patients is limited. Further research needs to be carried out for management and prophylaxis for patients with COVID-19 at risk of aortic thrombosis.

We present a case of a middle-aged man with multiple comorbidities and remote COVID-19 infection who came to the emergency room with signs and symptoms worrisome for a cerebrovascular accident (CVA). Brain imaging revealed multiple cortical infarcts suggestive of a cardioembolic etiology. During his hospitalization, he underwent a transesophageal echocardiogram (TEE) that showed a 1x1 cm mobile thrombus in the distal descending aorta. Laboratory workup was negative for any hypercoagulable condition; it was thought that this patient might have a hypercoagulable state post-COVID-19 infection. After a thorough risk vs. benefit discussion, patient was started on apixaban. He remains alive and is doing well in a recent follow-up in our clinic.

## Introduction

Severe acute respiratory syndrome coronavirus 2 (SARS-CoV-2) manifestations are unfolding with time, and several pathologies have been identified as possible sequelae of this infection. One such manifestation is a hypercoagulable state that presents as thrombosis/embolism [1]. The pathophysiology of thromboembolism is poorly understood [2-3]. We present one such case of a patient with aortic arch thrombus and thromboembolic cerebrovascular accident (CVA) who had a remote coronavirus disease 2019 (COVID-19) infection.

## Case presentation

A 57-year-old man with a past medical history of hypertension, hyperlipidemia, tobacco use, and recent COVID-19 infection (fully vaccinated with Pfizer) presented to the emergency room with complaints of slurred speech, left-sided facial droop, and left-sided arm weakness. 

In the emergency room, his vital signs revealed blood pressure of 167/97 mmHg, heart rate of 106 beats/minute, and oxygen saturation (SPO2) of 96% on room air. On physical exam, the patient was lying comfortably in bed speaking in complete sentences with slurred speech and left-sided facial droop, cranial nerves II-XII were intact. Left-sided upper arm strength was 4/5, the right arm was 5/5, and no pronator drift was noted. Lower extremities strength was 5/5 with intact sensation in both upper and lower extremities. Cardiovascular exam was significant for tachycardia, otherwise unremarkable. Stroke code was called, and the patient was immediately taken for non-contrast computed tomography (CT) of the head, which revealed no signs of intracranial hemorrhage. 

Initial laboratory work is shown in Table 1. Urine toxicology was negative.

**Table 1:** Initial Laboratory work-up. PCO2: partial pressure of carbon dioxide, INR: international normalized ratio

Test	Results	Reference Range
White Blood Count	14.4	5.00 - 11.00 x10E3/uL
Hemoglobin	14.8	12.0 - 15.0 G/DL
Platelet	285	150 - 400 x10E3/uL
Sodium	134	135 - 145 mmol/L
Potassium	4.0	3.5 - 5.2 mmol/L
Chloride	100	96 - 108 mmol/L
Phosphorus	4.7	2.4 - 4.7 mg/dL
Magnesium	1.9	1.5 - 2.5 mg/dL
Creatinine	1.24	0.5 - 1.1 MG/DL
Blood Urea Nitrogen	22	6 - 23 MG/DL
Brain Natriuretic Peptide	72.4	0.0 - 100 pg/mL
Troponin	0.01	<0.031 mg/dl
Aspartate aminotransferase	26	1 - 35 U/L
Alanine aminotransferase	36	1 - 45 U/L
Alkaline phosphatase	114	38 - 126 U/L
Bilirubin Direct	0.2	0.0 - 0.8 mg/dL
Bilirubin Total	0.8	0.1 - 1.2 mg/dL
Hemoglobin A1c	14.8	13.9 - 16.3 g/dL
Anion gap	23	7 - 16 mmoL/L
Ph	7.39	7.35 - 7.45
PCO2	33.2	35 - 45 mmHg
Bicarbonate	19.7	21 - 28 mEq/L
Lactic acid	2.75	0.50 - 2.00 mmol/L
Beta-Hydroxybutyrate	1.79 mmol/L	< 0.3 mmol/L
Urine Studies		
GLUCOSE:	>/=500	Negative mg/dL
KETONE	Trace	Negative mg/dL
LEUKOCYTE ESTERASE:	Negative	Negative
NITRITE	Negative	Negative
PROTEIN	30	Negative mg/dL
SPECIFIC GRAVITY	1.016	1.004 - 1.036
WBC NO./AREA URNS HPF	2	0 - 5 /HPF
RBC	5	0 - 3 /HPF
Hypercoagulation Workup:		
Activated Partial Thromboplastin (APTT)	34.2	24.6 - 34.7 secs
Prothrombin time	13.8	12.0 - 14.2 secs
INR	1.1	0.9 - 1.1
Lupus anticoagulant panel [Dilute Russell's viper venom time (dRVVT)]	37.4	27.0 - 41.0 sec
Antithrombin activity	110	81-113%
Protein C	148	75- 133%
Protein S	80	52 - 151%
Beta 2 glycoprotein antibodies (IGG, IGM, IGA)	<9	0 - 25 GPI IgA units
Anticardiolipin antibody	<9	0 - 14 GPL U/mL
Homocysteine level	7.0	<11 umol/L for both male and female
Fibrinogen Levels	769	212 - 516 mg/dL
D-Dimer	3.96	<0.5 ug/mL FEU
DRUGS OF ABUSE SCREEN		
Amphetamine Screen	Negative	< 1000 ng/mL
Benzodiazepine Screen	Negative	<200 ng/mL
Cannabinoid (THC) Screen	Negative	<50 ng/mL
Cocaine Screen	Negative	<300 ng/mL
Methadone Screen	Negative	<300 ng/mL
Opiate Screen	Negative	<300 ng/mL
Phencyclidine Screen	Negative	<25 ng/mL
Barbiturate Screen	Negative	<200 ng/mL

**Figure 1:**
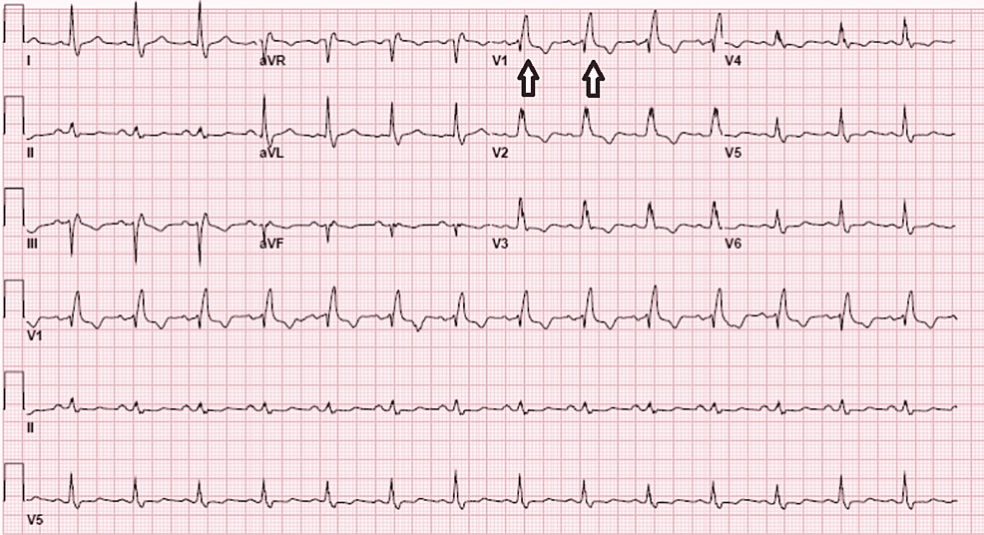
RSR’ pattern in identified as white arrows in lead V1 (“M-shaped” QRS complex)

The patient was given intravenous (IV) tenecteplase which caused a mild improvement of the neurological deficits. However, the patient became acutely diaphoretic and had multiple episodes of nonbilious and non-bloody emesis. Suddenly he became less responsive, somnolent, with right gaze preference, worsening left hemiparesis and left facial droop. The patient was taken to repeat head CT with no evidence of bleeding again. An electrocardiogram (EKG) revealed a new right bundle branch block (RBBB) (Figure 1).

**Figure 2:**
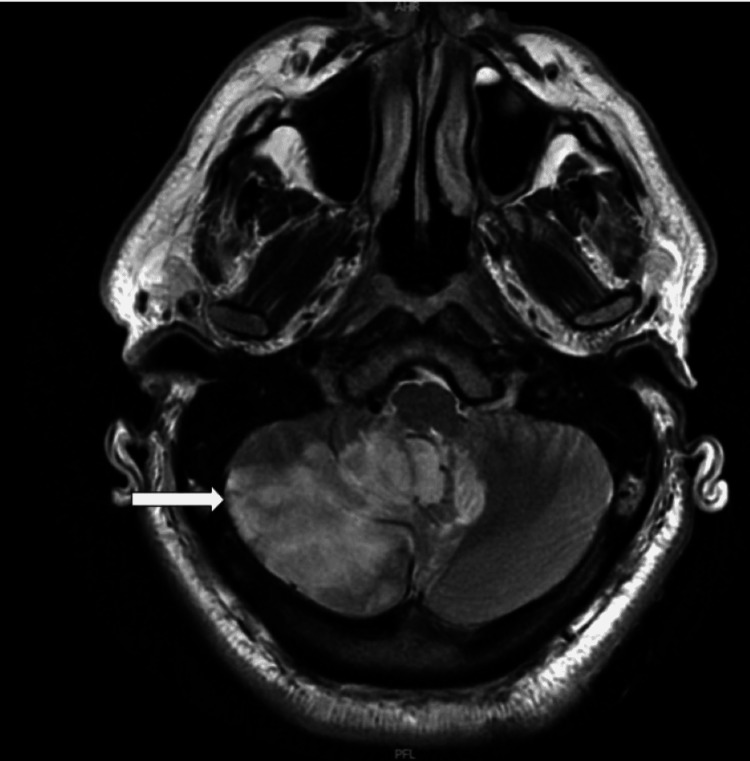
Right sided hyperintense lesion in the cerebellum with left shift in brain magnetic resonance imaging (MRI) Brain magnetic resonance imaging (MRI) performed with axial plane utilizing gradient echo sequence which revealed right sided hyperintense lesion in the cerebellum with minimal midline shift. "

He was admitted to the intensive care unit (ICU). Laboratory workup was also notable for diabetic ketoacidosis, and the patient was started on an insulin drip in the ICU, which was later bridged to long-acting insulin after the anion gap normalized. The CT head was repeated, and it revealed subacute right frontal, right parietal, and right cerebellar infarcts consistent with right posterior inferior cerebellar artery infarct, with mass effect evident with mild compression of brainstem, fourth ventricle compression with signs of early hydrocephalus. Bilateral lower extremity doppler was negative. Magnetic resonance imaging revealed (MRI) right-sided infarcts (Figure 2) with posterior fossa mass effect, signs of early hydrocephalus, and hemorrhagic conversion.

The patient was continuously lethargic in the ICU with worsening orientation (awake, alert, and oriented to person and place [AAOx2]), still complaining of nausea and inability to tolerate anything orally. The neurosurgery team was consulted, given concerns of worsening mental status changes, and a new hydrocephalus. Patient underwent sub-occipital craniectomy with external ventricular drain (EVD) placement under general anesthesia. The hospital course was complicated by new onset of fevers and chest x-ray with new opacities worrisome for ventilator-associated pneumonia; the patient was started on vancomycin and cefepime and was later found to be COVID-19 positive on the fifth day of admission. He underwent a transesophageal echocardiogram (TEE) which revealed a 1x1 cm mobile thrombus in the distal descending aorta. 

Hypercoagulation workup was ordered and revealed results as mentioned in Table 1. Also, laboratory workup was negative for factor 5 Leiden and factor 2 gene mutation. The CVA was attributed to remote infection from COVID-19 after ruling out the other secondary causes of hypercoagulability.

The patient's hospital course was further complicated by pulseless electrical activity (PEA) cardiac arrest with return of spontaneous circulation (ROSC) after six minutes. Later found to have significant mucous plugging on bronchoscopy, which was successfully removed. A repeat computed tomography angiogram (CTA) of the chest revealed small filling defects in both the ascending and descending thoracic aorta in correlation to the aortic thrombus.

Cardiothoracic surgery (CTS) was consulted and deemed the patient unstable for surgical intervention. Given the significant risk of hemorrhagic conversion with recent CVA, multiple specialties were consulted to decide whether to start the patient on anticoagulation. After a risk vs. benefit discussion, it was decided to start the patient on a heparin drip which was later switched to apixaban 5mg two times daily. 

His condition continued to improve; the patient completed his antibiotic treatment after observation on the medicine floors; he was deemed stable and was discharged home on apixaban 5mg two times daily. In his follow-up visits, the patient was seen to be healthy and doing well. 

## Discussion

SARS-CoV-2 has been associated with multiple manifestations; one manifestation that has been frequently encountered, unrelated to the severity of infection, is the increased risk of arterial and venous thrombosis [1]. Patients are at high risk of developing deep venous thrombosis (DVT) and pulmonary embolism [1-2]. Pulmonary embolism is the most common form of thromboembolism in patients with COVID-19 infection. In some studies, the incidence of pulmonary thrombosis was as high as 79% [1]. A large study was conducted in the Netherlands on COVID-19 patients admitted to the ICU. It was found that the incidence of venous thromboembolism was 27% in patients who were on standard DVT prophylaxis and the incidence of pulmonary embolism was 81% [2-6].

SARS-COV-2 has four proteins that play an essential role in infecting the host. These proteins are spike, membranous, envelope, and nuclear capsid proteins. The virus enters the host cell with the help of spike protein; it binds to the angiotensin-converting enzyme 2 (ACE-2) receptor, which is expressed by type II pneumocytes, vascular endothelial cells, and cardiac myocytes [1-2]. Various mechanisms have been proposed for the pathophysiology of hypercoagulable state and COVID-19 infection. The virus itself directly damages the vascular endothelium resulting in microvascular thrombi formation. It also activates the complement pathway, and these complement deposits into the endothelium, resulting in microvascular thrombi formation [1]. COVID-19 infection also leads to the release of pro-inflammatory cytokine production, including tumor necrosis factor-alpha, interleukin-1, and interleukin-6. The cytokines, particularly interleukin-6, increase the expression of tissue factors, which results in activation of extended coagulation positive and leads to the formation of thrombin and fibrin, which ultimately resulted in thrombus formation.

Although we now have some data for the incidence of DVT and pulmonary embolism in patients with COVID-19 infection, the data for aortic thrombus is scarce. Due to the scarcity of the data, it is hard to predict the exact incidence of aortic thrombosis in patients infected with COVID-19 [7]. The current data on aortic thrombosis in patients with COVID-19 infection is limited to the case report and case series. In one literature review, 43 cases of COVID-19-related aortic thrombosis were identified, and most of the patients were male and above age 50 [7]. The common comorbidities were smoking, obesity, hypertension, and diabetes mellitus [7-8].

The patient with aortic thrombosis can present with acute limb ischemia, CVA, chest pain, and abdominal pain [7-8]. A large number of patients were also found to have incidental aortic thrombosis [7]. So the diagnosis of aortic thrombosis should be considered in any patient of COVID-19 infection who presents with abdominal pain, chest pain, motor weakness, sensory loss, vision loss, facial drop, slurred speech, limb ischemia, or other signs of end-organ damage, particularly if they have elevated D-dimer as the higher D-dimer level is associated with increased mortality [7]. 

**Figure 3:**
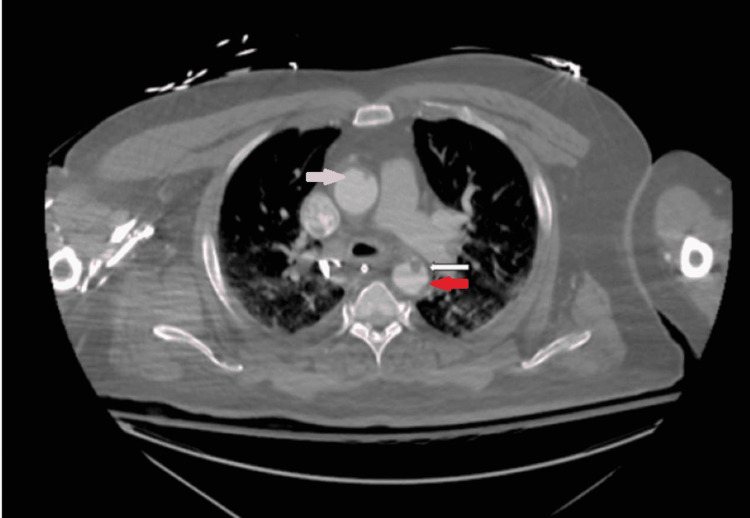
Computed tomography angiogram (CTA) of the chest revealed small filling defects in both the ascending (pink arrow) and descending thoracic aorta (red arrow), highlighted by white arrow.

**Figure 4:**
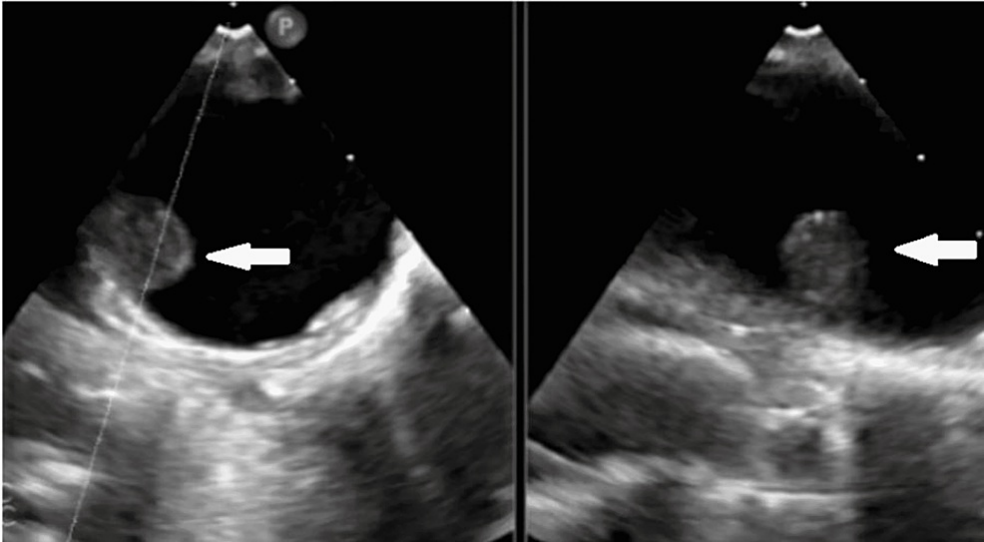
Transesophageal Echocardiogram (TEE) 1x1 cm mobile thrombus at the junction of the distal aortic arch-thoracic aorta.

The diagnosis of aortic thrombosis is confirmed with CTA. The TEE, magnetic resonance angiogram, and intravascular ultrasound can be considered for diagnosing patients with CTA contraindication, such as renal failure [7]. CTA of our patient revealed filling defects (Figure 3). TEE findings confirmed a mobile thrombus in the descending aorta (Figure 4).

Given the rarity of the disease and the absence of randomized control trials, there are no specific treatment guidelines for the management of aortic thromboembolism. The treatment options include anticoagulation, surgical/endovascular thrombectomy, and thrombolysis [7]. Unfortunately, no head-to-head trials comparing medical versus surgical management of aortic thrombosis in patients with COVID-19 infection. It is recommended to start anticoagulation (if there is no contraindication) in all patients with aortic thrombosis to prevent thrombus progression once the diagnosis is confirmed [7]. And the multidisciplinary team should be involved in formulating the treatment plan on a case-by-case basis.

Our patient was tested negative for COVID-19 on presentation and vaccinated with Pfizer twice. Patient had images confirming ischemic infarcts and TEE revealing a distal aortic thrombus. Laboratory workup was negative for any hypercoagulable conditions such as antiphospholipid syndrome, Factor 5 Leiden gene mutation, Prothrombin gene mutation, deficiencies of natural proteins that prevent clotting- such as antithrombin, protein C, and protein S, negative for elevated levels of homocysteine. To note this patient's fibrinogen level was mildly elevated which was collected after he was found to have a COVID-19 infection that he contracted in the hospital, likely an acute phase reaction. After a thorough workup was negative, a remote infection with COVID-19 and thrombosis in different organ systems, it was suggested that this patient might have a hypercoagulable state post-COVID-19 infection. A dilemma arose when the patient was supposed to start anticoagulation with recent CT findings concerning hemorrhagic conversion. After a thorough risk vs benefit discussion the patient was started on anticoagulation with discharge and follow up as mentioned above. 

## Conclusions

Post-COVID-19 hypercoagulable states are increasingly being recognized. Aortic thrombosis is a rare complication of COVID-19 infection. The timely diagnosis with the help of CTA is vital to prevent catastrophic complications such as myocardial ischemia, thromboembolic stroke, limb ischemia, and mesenteric ischemia. The management of aortic thrombosis in COVID-19 patients can be challenging given the rare disease and the absence of randomized control trials. According to the facts pertaining to the particular situation, the management varies on a case-by-case basis. This case also emphasized the need for further studies to establish a clear guideline for the management of aortic thrombosis in patients with COVID-19 infection.

## References

[1] Kichloo A, Dettloff K, Aljadah M (2020). COVID-19 and hypercoagulability: a review. Clin Appl Thromb Hemost.

[2] Abou-Ismail MY, Diamond A, Kapoor S, Arafah Y, Nayak L (2020). The hypercoagulable state in COVID-19: incidence, pathophysiology, and management. Thromb Res.

[3] Mortus JR, Manek SE, Brubaker LS, Loor M, Cruz MA, Trautner BW, Rosengart TK (2020). Thromboelastographic results and hypercoagulability syndrome in patients with coronavirus disease 2019 who are critically ill. JAMA Netw Open.

[4] Whyte CS, Morrow GB, Mitchell JL, Chowdary P, Mutch NJ (2020). Fibrinolytic abnormalities in acute respiratory distress syndrome (ARDS) and versatility of thrombolytic drugs to treat COVID-19. J Thromb Haemost.

[5] Zuin M, Rigatelli G, Zuliani G, Roncon L (2021). The risk of thrombosis after acute-COVID-19 infection. QJM.

[6] Han H, Yang L, Liu R (2020). Prominent changes in blood coagulation of patients with SARS-CoV-2 infection. Clin Chem Lab Med.

[7] Karabulut K, Kapici A, Andronikashvili A, Morgan J (2021). A review of aortic thrombosis in COVID-19 infection. Explor Med.

[8] Wickham H, Tam JC, Chan XH, George MJ, Levi M, Brown M (2021). Aortic thrombosis in COVID-19. Clin Infect Pract.

